# Intravitreal Anti-vascular Endothelial Growth Factor Injection for Retinopathy of Prematurity: A Systematic Review and Meta-Analysis

**DOI:** 10.3389/fmed.2022.884608

**Published:** 2022-05-09

**Authors:** Nada O. Taher, Abdullah A. Ghaddaf, Sarah A. Al-Ghamdi, Jumanah J. Homsi, Bandar J. Al-Harbi, Lugean K. Alomari, Hashem S. Almarzouki

**Affiliations:** ^1^College of Medicine, King Saud bin Abdulaziz University for Health Sciences, Jeddah, Saudi Arabia; ^2^King Abdullah International Medical Research Center, Jeddah, Saudi Arabia; ^3^Ophthalmology Saudi Board Program, Jeddah Eye Hospital, Jeddah, Saudi Arabia; ^4^College of Medicine, King Abdulaziz University, Jeddah, Saudi Arabia; ^5^Ophthalmology Saudi Board Program, King Abdulaziz University Hospital, Jeddah, Saudi Arabia; ^6^Department of Ophthalmology, Ministry of the National Guard-Health Affairs, Jeddah, Saudi Arabia

**Keywords:** retinopathy of prematurity, anti-vascular endothelial growth factor, bevacizumab, ranibizumab, laser photocoagulation

## Abstract

**Background:**

Laser photocoagulation and/or intravitreal anti-vascular endothelial growth factor (anti-VEGF) injections constitute the current standard treatment for retinopathy of prematurity (ROP). This systematic review and meta-analysis aimed to assess the efficacy and safety of anti-VEGF monotherapy for ROP treatment using the Grading of Recommendations Assessment, Development and Evaluation (GRADE) approach.

**Methods:**

We searched the Medline, Embase, and Cochrane Central Register of Controlled Trials (CENTRAL) databases. We included randomized controlled trials (RCTs) that compared intravitreal anti-VEGF monotherapy (e.g., bevacizumab, ranibizumab, aflibercept, and pegaptanib) with laser photocoagulation in preterm infants with ROP. We evaluated the rates of recurrence, treatment switching, retreatment, adverse events, and mortality. The risk ratio (RR) was used to represent dichotomous outcomes. Data were pooled using the inverse variance weighting method. The quality of evidence was assessed using the GRADE approach. Risk of bias was assessed using the Revised Cochrane risk of bias tool for randomized trials.

**Results:**

Seven RCTs (*n* = 579; 1,158 eyes) were deemed eligible. Three RCTs had an overall low risk of bias, three had some concerns, and one had an overall high risk of bias. The pooled effect estimate showed a statistically significant reduction in adverse events in favor of anti-VEGF monotherapy [RR = 0.17, 95% confidence interval (CI) 0.07–0.44]. The pooled analysis showed no significant difference between the anti-VEGF and laser groups in terms of recurrence rate (RR = 1.56, 95% CI 0.23–10.54), treatment switching (RR = 2.92, 95% CI 0.40–21.05), retreatment (RR = 1.56, 95% CI 0.35–6.96), and mortality rate (RR = 1.28, 95% CI 0.48–3.41).

**Conclusion:**

Overall, intravitreal anti-VEGF monotherapy was associated with fewer adverse events than laser therapy, rated as high quality of evidence according to the GRADE criteria. Pooled analysis revealed no significant difference between the two arms with respect to the recurrence rate, treatment switching, retreatment, and mortality rate, with quality of evidence ranging from moderate to very low as per the GRADE approach.

**Systematic Review Registration:**

[https://www.crd.york.ac.uk/prospero/#recordDetails], identifier [CRD42021270077].

## Introduction

Retinopathy of prematurity (ROP), formerly known as retrolental fibroplasia, is a common cause of preventable blindness in children (1). ROP is a neovascular disorder caused by reduced retinal vascularization in premature infants (2). Annually, around 32,000 neonates develop ROP-induced blindness or severe visual impairment worldwide; ROP mostly occurs in infants with a gestational age ≤ 30 weeks or birth weight ≤ 1,500 g (3, 4). Thus, screening for ROP among premature infants is commenced to identify ROP that requires therapeutic intervention as early as possible (5).

In recent decades, the standard treatment for ROP was cryotherapy. Nowadays, laser photocoagulation and intravitreal anti-vascular endothelial growth factor (anti-VEGF) injections have completely replaced cryotherapy and become the new standard treatment for ROP (1, 6). The treatment choice mainly relies on the experience and preference of the treating ophthalmologist and the preference of the patients’ guardians (7–10).

Despite many studies encouraging the use of anti-VEGF agents, the long-term outcomes of anti-VEGF therapy, optimal frequency and duration of follow-up, and optimal management of recurrence remain unclear (6, 11). Additionally, no previous systematic review has described the roles of different anti-VEGF agents or the management of different ROP zones.

This systematic review and meta-analysis aimed to comprehensively describe the efficacy and safety of intravitreal anti-VEGF injection with bevacizumab, ranibizumab, aflibercept, or pegaptanib and compare it with retinal ablative therapy for ROP management in terms of recurrence, treatment switching, retreatment, adverse events, and mortality.

## Methods

This systematic review was conducted in accordance with a pre-specified protocol registered with PROSPERO (CRD42021270077) and conformed with the Preferred Reporting Items for Systematic Reviews and Meta-Analysis (PRISMA) checklist (12).

### Eligibility Criteria

We reviewed randomized controlled trials (RCTs) that included preterm infants with ROP who underwent either intravitreal anti-VEGF monotherapy or laser photocoagulation and collected information on the following pre-specified outcomes: recurrence rate, treatment switching (i.e., the need for a treatment modality other than that assigned), retreatment, adverse events, and mortality rate. We excluded trials that enrolled participants with previous operative or non-operative management of ROP and those that included participants with vitreoretinal conditions other than ROP. The outcomes of retreatment and treatment switching were investigated as two separate outcomes instead of combining them as one, named “additional treatment”. Such distinction was made due to the reasons behind each of them. That is, retreatment is usually done in lack of adequate regression of ROP after treatment (5). Some RCTs considered retreatment approach once ROP recurrence occurs (1, 11). On the other hand, treatment switching is done mostly due to developing complications that are specific to the assigned treatment modality. For example, anti-VEGF agents are not injected in participants with signs of conjunctival infection (5). Additionally, treatment switching is sometimes used for the sake of trying a different approach in the management of ROP (1, 11). Nevertheless, there was inconsistency in some of the enrolled RCTs regarding the reasons for retreatment or treatment switching as well as a lack of reporting the reasons behind using an additional treatment. Therefore, by separating these two outcomes in our review, we aimed to evaluate the effectiveness of using an additional treatment (e.g., retreatment and treatment switching) in the cases of ROP persistence or recurrence as well as see the trends in the additional treatment, whether using the same treatment modality or switching to another modality.

### Search Strategy

We systematically searched the Medline, Embase, and Cochrane Central Register of Controlled Trials (CENTRAL) databases from database inception to July 15, 2021, without any restriction on date or language. The complete search strategy is provided in the Supplementary Appendix. We manually searched the references of the included studies for potentially relevant RCTs that were missed during the systematic search.

### Study Selection and Data Extraction

Two reviewers, independently and together, performed title and abstract screening against the eligibility criteria, full-text assessment, and data extraction from eligible trials. Discrepancies were resolved through consensus or discussion with a third reviewer before performing analyses.

### Meta-Analysis

Data analysis was performed using RevMan (Review Manager) version 5.3 (Cochrane Collaboration). All statistical analyses were performed using the random-effects model. We adopted 95% as a confidence level and *P* < 0.05 as a threshold. The statistical heterogeneity was assessed using I^2^ and the *P*-value of the Chi-square test. Dichotomous outcomes (recurrence rate, treatment switching, retreatment, adverse events, and mortality rate) were expressed as risk ratios (RRs) and pooled using the inverse variance weighting method. We performed subgroup analysis based on the following zones: zone I, zone II, and undetermined zone. The undetermined zone subgroup comprised two RCTs (Stahl et al. and O’Keeffe et al.) in which anti-VEGF monotherapy [e.g., intravitreal ranibizumab (IVR) and intravitreal bevacizumab (IVB)] were compared with laser therapy without specifically considering the ROP zone (10, 13). Instead of excluding these studies, we added an undetermined zone subgroup. Although these studies do not provide insight into the effects by the ROP zone, their findings have considerable weightage in the pooled effect estimate of each outcome and improve the power of our study. The quality of evidence for each outcome was assessed using the Grading of Recommendations Assessment, Development and Evaluation (GRADE) criteria.

### Risk of Bias Assessment

Two reviewers, independently and together, used the Revised Cochrane risk of bias tool to assess the risk of bias in the eligible RCTs (14). Each study was reviewed and scored as high risk, low risk, or some concerns. Discrepancies between the reviewers were resolved through discussion until agreement. We assessed the potential for publication bias for each outcome *via* visual inspection of a funnel plot with the RR and standard error. Evidence of publication bias was considered possible when the funnel plot was asymmetrical.

## Results

**FIGURE 1:**
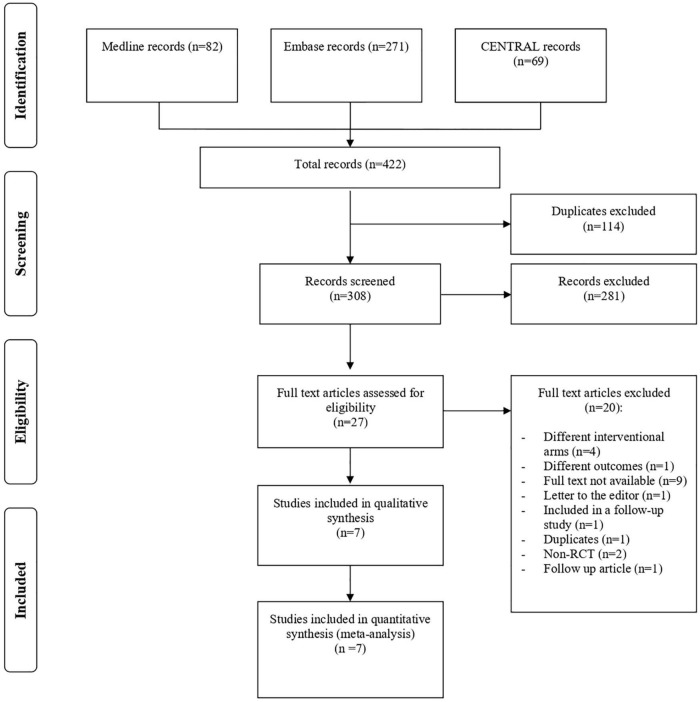
Study flow diagram. CENTRAL, Cochrane Central Register of Controlled Trial; RCT, randomized controlled trial.

Figure 1 shows the flowchart of study inclusion, with justifications for excluding studies. From the literature search, we identified 422 articles, of which 114 duplicates were excluded. After examining the titles and abstracts, 27 potentially eligible studies were assessed for inclusion. Eventually, seven RCTs were deemed eligible and included in the meta-analysis. Five RCTs assessed IVB and two evaluated IVR. No RCTs were found on aflibercept or pegaptanib monotherapy.

### Trial Characteristics

This meta-analysis included 579 infants (1,158 eyes) (1, 3, 10, 11, 13, 15, 16). Of them, 267 (534 eyes), 213 (426 eyes), and 99 (198 eyes) were randomly assigned to the laser therapy, IVB monotherapy, and IVR groups, respectively. The mean gestational age ranged from 24.2 to 28.96 weeks for the arm of anti-VEGF monotherapy and from 24.3 to 28.50 weeks for the arm of laser therapy. The mean birth weight ranged from 615.20 to 1,232 g for anti-VEGF group and from 657.90 to 1,273 g for the laser group. Table 1 shows the detailed characteristics of the included studies.

**TABLE 1:** Characteristics of the included studies.

Gender		The definition of ROP requiring treatmen	Measured outcomes	Mean birth weight (g)	Gestational age (weeks)			Number of participants		Number of eyes		Anti-VEGF dose	Intervention	Author, year
Male	Female			Anti-VEGF	Laser	Anti-VEGF	Anti-VEGF dose	Laser	Anti-VEGF	Laser	Anti-VEGF	Laser		
39	40	Zone II (stage 2 or 3 ROP with plus disease).	Recurrence, treatment switch, retreatment, adverse events, and death.	28.37 (±1.96)	1202 (±321)	1133	0.625 mg/0.025 mL	36	43	72	86	28.50 (±1.99)	Intravitreal bevacizumab vs. Laser	Karkhaneh (1)
NR	NR	Zone 1 (stage 3 with/without plus disease).	Structural changes and retinal and choroidal findings on fluorescein angiogram and digital retinal photographs 9 months and 4 years after treatment.	4 infants: mean gestational age = 25.3 (range 22.7-29.3). 17 infants; mean gestational age = 25.6 (range 22.7-29.3).	4 infants: 697 (range 615-755). 17 infants: 667 (range 380-960).	4 infants: 697 (range 615-755). 17 infants: 667 (range 380-960).	0.5mg/0.02mL	21		21	21	4 infants: mean gestational age = 25.3 (range 22.7-29.3). 17 infants; mean gestational age = 25.6 (range 22.7-29.3).	Intravitreal bevacizumab vs. Laser	Lepore (13)
97	53	Zone I or zone II posterior (stage 3 with plus disease).	Recurrence, adverse events, and death.	Zone I ROP: 24.2 Zone II ROP: 24.5	Zone I ROP: 657.9 Zone II ROP: 680.7	Zone I ROP: 615.2 Zone II ROP: 689.2	0.625mg/0.025 mL	75	75	150	150	Zone I ROP: 24.3 Zone II ROP: 24.5	Intravitreal bevacizumab vs. Laser	Mintz-Hittner (3)
NR	NR	Zone I or posterior zone II with plus disease.	Recurrence, treatment switch, retreatment, and death.	Median (range): 25 (24-29)	Median (range): 780 (540-1080)	Median (range): 780 (540-1080)	1.25 mg/0.05mL	15		15	15	Median (range): 25 (24-29)	Intravitreal bevacizumab vs. Laser	O’Keeffe (14)
NR	NR	Zone II (stage 2 or 3 with plus disease).	Regression, treatment switch retreatment, adverse events, and death.	28.75 (±1.86)	1273 (±273)	1232 (±318)	0.625mg/0.025 mL	39	77	78	154	28.32 (±2.11)	Intravitreal bevacizumab vs. Laser	Roohipoor (15)
107	118	Zone I (stage 1, 2 or 3 2 with plus disease), Zone II (stage 3 with plus disease), or aggressive posterior retinopathy of prematurity.	Treatment switch, retreatment, adverse events, and death.	0.1 mg median (range): 26 (23-32) 0.2 mg median (range): 25 (23-32)	831 (±284)	0.1 mg: 886 (±299) 0.2 mg: 791 (±244)	0.1 mg 0.2 mg	74	74	148	148	Median (range): 26 (23-32)	Intravitreal ranibizumab vs. Laser	Stahl (9)
28	22	Zone II (Stage 2 or 3 with plus disease).	Recurrence, treatment switch, retreatment, and adverse events.	28.96 (±1.59)	1.06 (±0.24) (kg)	1.22 (±0.32) (kg)	0.3 mg/0.03 mL	25	75	50	50	28.27 (±1.84)	Intravitreal ranibizumab vs. Laser	Zhang (10)

*NR, not reported; ROP, retinopathy of prematurity; VEGF, vascular endothelial growth factor.*

### Risk of Bias Assessment

**FIGURE 2:**
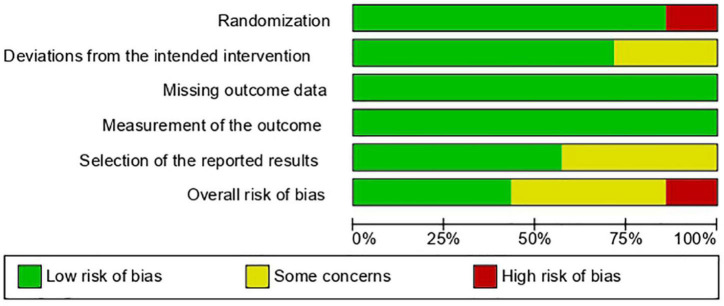
Risk of bias graph.

**FIGURE 3:**
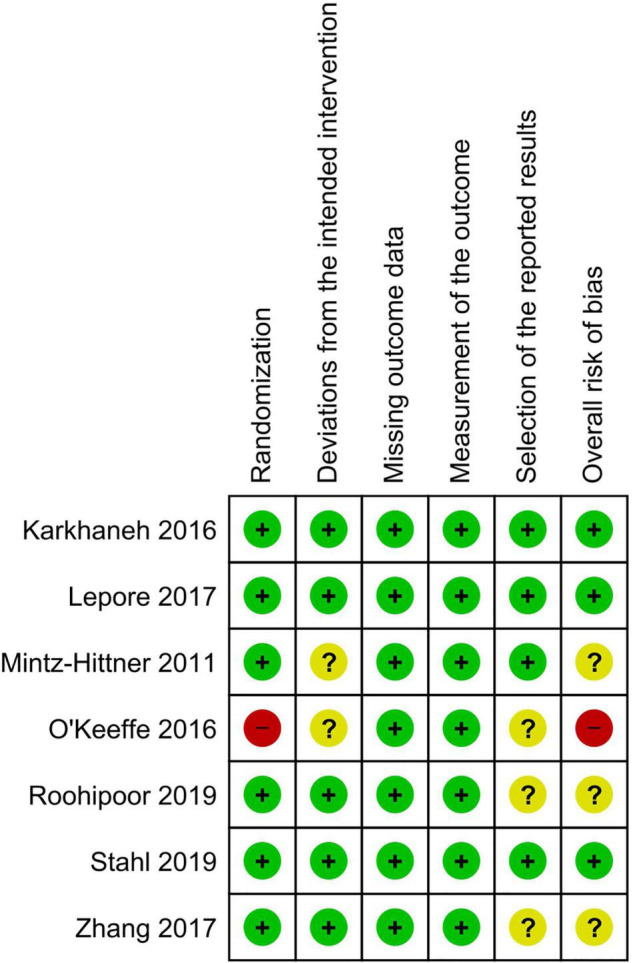
Risk of bias summary.

Three of the seven RCTs had an overall low risk of bias, three had some concerns, and one had an overall high risk of bias due to an issue with the randomization technique. Figures 2, 3 show the risk of bias assessment of the included RCTs.

### Recurrence Rate

**FIGURE 4:**
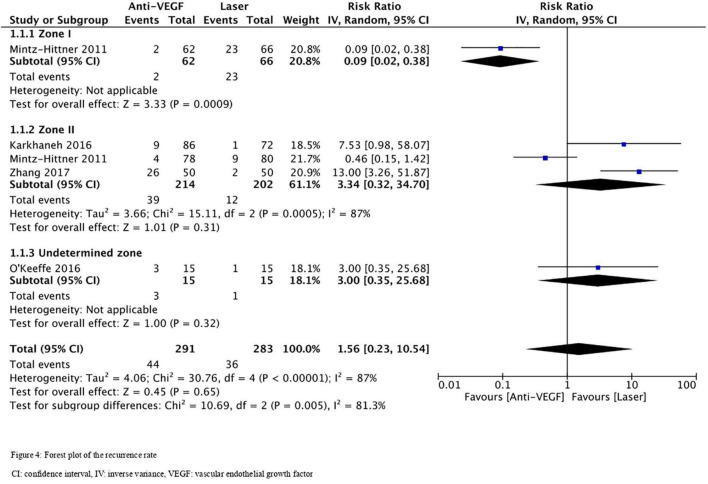
Forest plot of the recurrence rate. CI, confidence interval; IV, inverse variance; VEGF, vascular endothelial growth factor.

**FIGURE 5:**
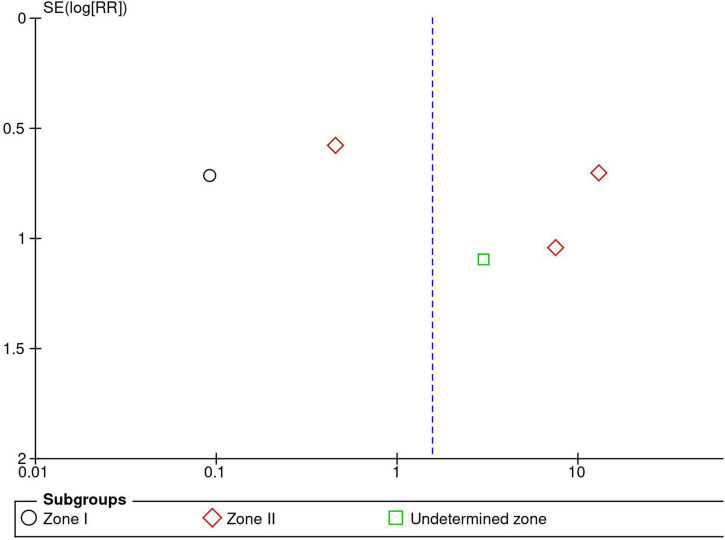
Funnel plot of recurrence rate. SE, standard error; RR: risk ratio.

**FIGURE 6:**
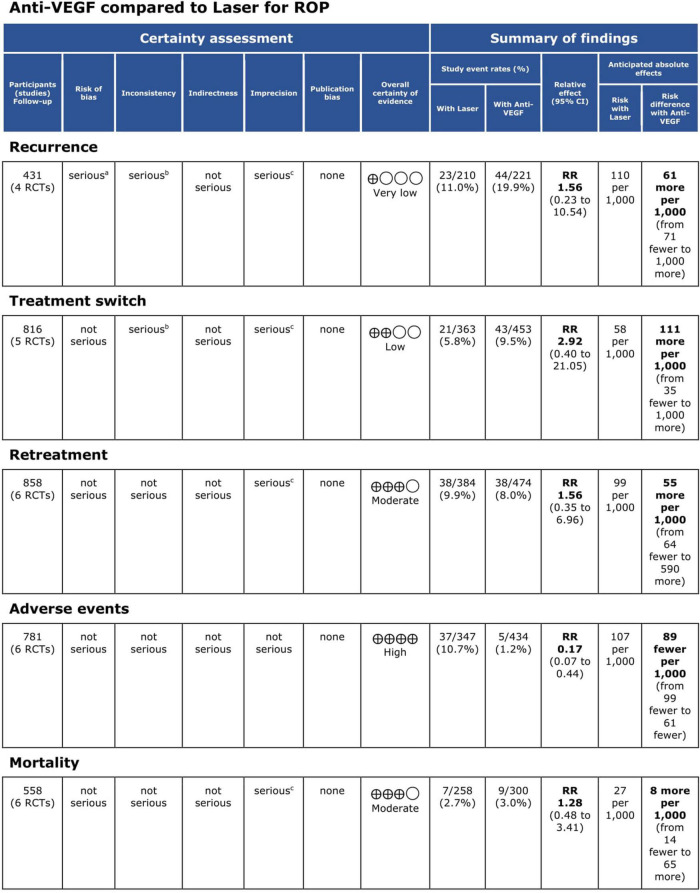
Grading of Recommendations Assessment, Development and Evaluation (GRADE) evidence profile. CI, confidence interval; RCT, randomized controlled trial; ROP, retinopathy of prematurity; RR, risk ratio; VEGF, vascular endothelial growth factor.

Five RCTs (*n* = 820 eyes) reported data on ROP recurrence (1, 3, 11, 13, 16). No significant difference was noted between anti-VEGF monotherapy and laser photocoagulation therapy in recurrence rate (RR = 1.56, 95% CI 0.23–10.54, *P* = 0.65, I^2^ = 87%). The heterogeneity was 87%, indicating considerable variability in the data, which was mostly attributed to the Mintz-Hittner et al. trial (3). Subgroup analysis showed a significantly higher recurrence rate in the laser group than the anti-VEGF group at zone I (RR = 0.09, 95% CI 0.02–0.38, *P* < 0.001, I^2^ = not applicable). In contrast, no significant difference was observed between intravitreal anti-VEGF injections and retinal ablative therapy in the zone II (RR = 3.34, 95% CI 0.32–34.70, *P* = 0.31, I^2^ = 87%) and the undetermined zone (RR = 3.00, 95% CI 0.35–25.68, *P* = 0.32, I^2^ = not applicable) subgroups (Figure 4). The funnel plot was symmetric upon visual inspection; therefore, publication bias was unlikely (Figure 5). The GRADE certainty of evidence was found to be rated as very low for the rate of recurrence (Figure 6).

### Treatment Switching

**FIGURE 7:**
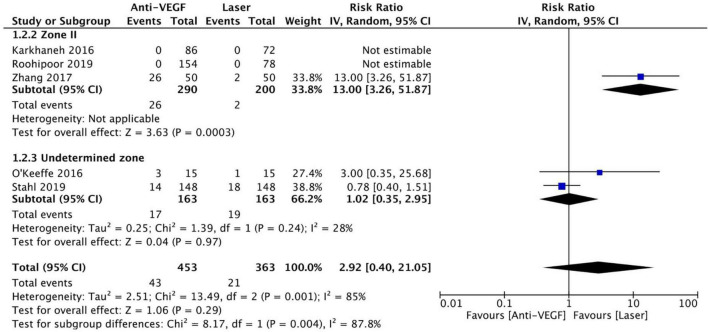
Forest plot of treatment switching. CI, confidence interval; IV, inverse variance; VEGF, vascular endothelial growth factor.

**FIGURE 8:**
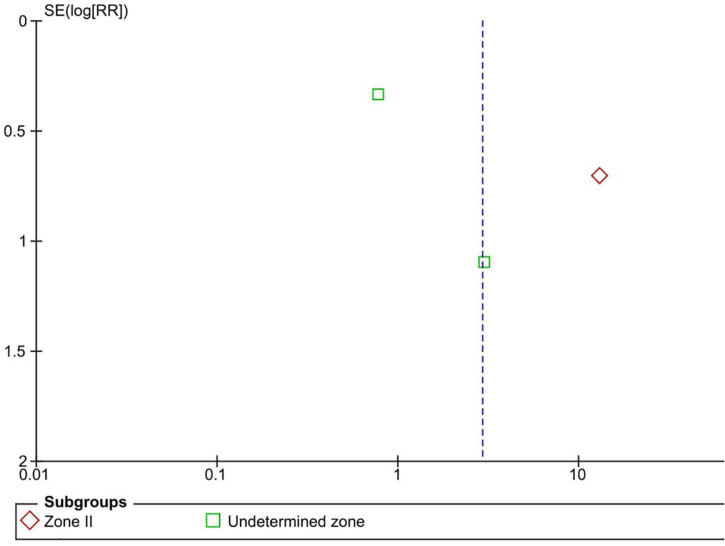
Funnel plot of treatment switching. SE, standard error; RR, risk ratio.

Five RCTs (*n* = 816 eyes) reported data on treatment switching (1, 10, 11, 13, 15). Intravitreal anti-VEGF injections and laser photocoagulation treatment showed similar treatment switching rates (RR = 2.92, 95% CI 0.40–21.05, *P* = 0.29, I^2^ = 85%). Subgroup analysis showed a significantly higher treatment switching rate in the anti-VEGF group than the laser group at zone II (RR = 13.00, 95% CI 3.26–51.87, *P* < 0.001, I^2^ = not applicable), but the treatment switching rates were comparable between the groups in the undetermined zone subgroup (RR = 1.02, 95% CI 0.35–2.95, *P* = 0.97, I^2^ = 28%). None of the included RCTs assessed treatment switching in patients with zone I ROP (Figure 7). No evidence of asymmetry was noted upon visual inspection of the funnel plot (Figure 8). The GRADE certainty of evidence was found to be rated as low for treatment switching (Figure 6).

### Retreatment

**FIGURE 9:**
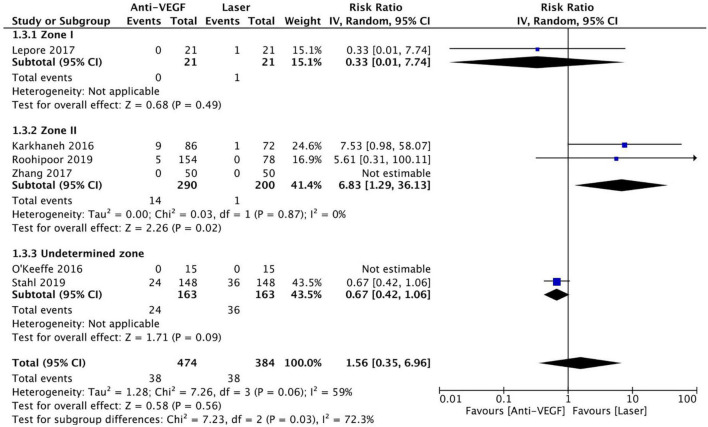
Forest plot of retreatment. CI, confidence interval; IV, inverse variance; VEGF, vascular endothelial growth factor.

**FIGURE 10:**
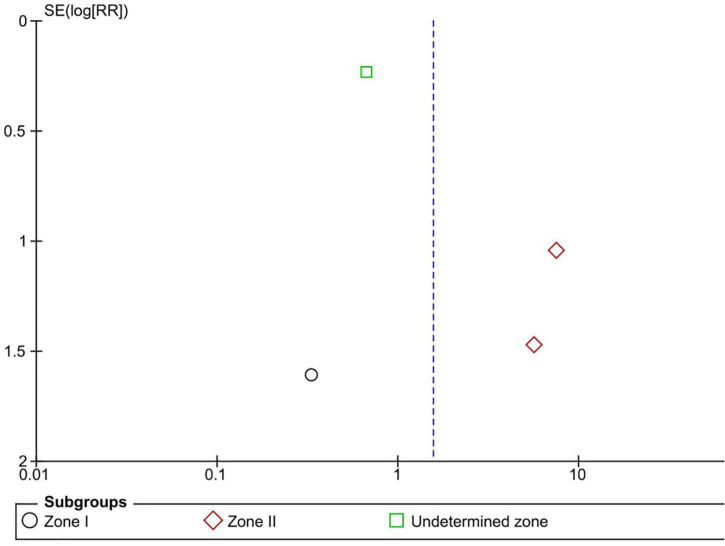
Funnel plot of retreatment. SE, standard error; RR: risk ratio.

Six RCTs (*n* = 900 eyes) reported data on retreatment (1, 10, 11, 13, 15, 16). Anti-VEGF injection and laser therapy showed similar retreatment rates (RR = 1.56, 95% CI 0.35–6.96, *P* = 0.56, I^2^ = 59%). No significant differences were noted between anti-VEGF injection and laser therapy in the zone I (RR = 0.33, 95% CI 0.01–7.74, *P* = 0.49, I^2^ = not applicable) and undetermined zone (RR = 0.67, 95% CI 0.42–1.06, *P* = 0.09, I^2^ = not applicable) subgroups. In contrast, the anti-VEGF group had a significantly higher retreatment rate than the laser group at zone II (RR = 6.83, 95% CI 1.29–36.13, *P* = 0.02, I^2^ = 0) (Figure 9). The funnel plot was symmetric upon visual inspection; therefore, publication bias was unlikely (Figure 10). The GRADE certainty of evidence was found to be rated as moderate for retreatment (Figure 6).

### Adverse Events

**FIGURE 11:**
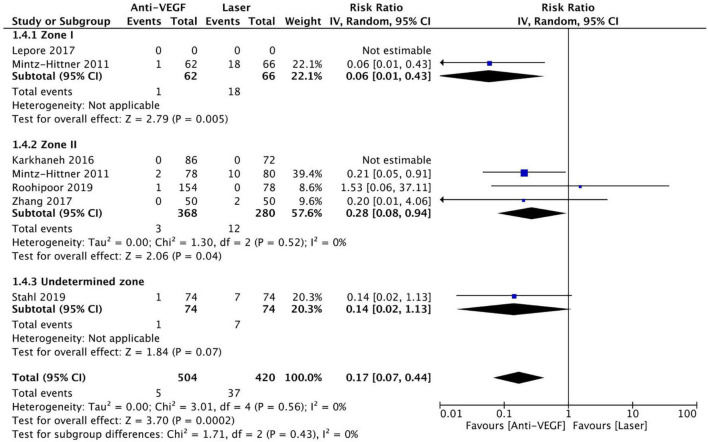
Forest plot of adverse events. CI, confidence interval; IV, inverse variance; VEGF, vascular endothelial growth factor.

**FIGURE 12:**
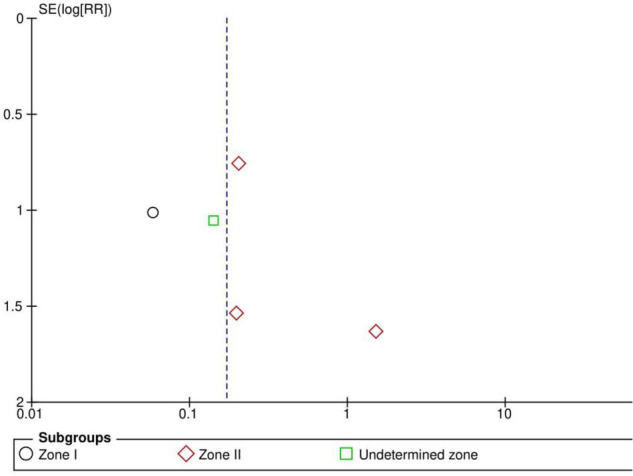
Funnel plot of adverse events. SE, standard error; RR, risk ratio.

Six RCTs (*n* = 1,170 eyes) reported data on adverse events (1, 3, 10, 11, 15, 16). Overall, intravitreal anti-VEGF injection showed a significantly lower adverse event rate than retinal ablative therapy (RR = 0.17, 95% CI 0.07–0.44, *P* < 0.001, I^2^ = 0%); myopic changes and unfavorable structural outcomes, such as macular ectopia and retinal folds, vitreous and retinal hemorrhages, and retinal detachment, were prevalent among the laser group (10, 11, 16). Similarly, a significantly higher adverse event rate was noted in the laser group than in the anti-VGEF injection group at zone I (RR = 0.06, 95% CI 0.01–0.43, *P* = 0.005, I^2^ = not applicable) and zone II (RR = 0.28, 95% CI 0.08–0.94, *P* = 0.04, I^2^ = 0%), but there was no significant difference between anti-VEGF injection and laser therapy in the undetermined zone subgroup (RR = 0.14, 95% CI 0.02–1.13, *P* = 0.07, I^2^ = not applicable) (Figure 11). No evidence of asymmetry was noted upon visual inspection of the funnel plot (Figure 12). The GRADE certainty of evidence was found to be rated high for adverse events (Figure 6).

### Mortality

**FIGURE 13:**
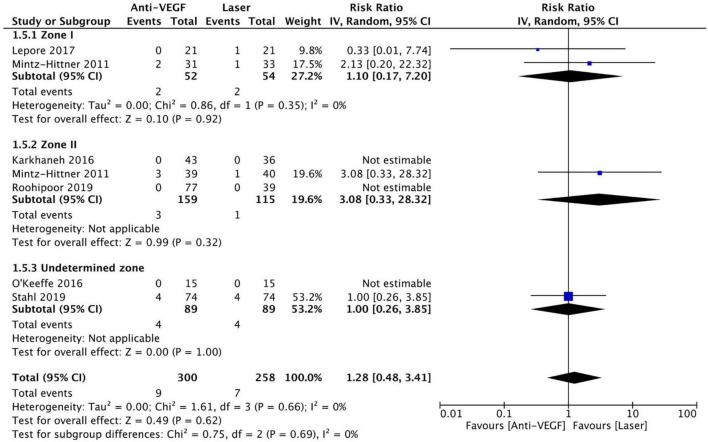
Forest plot of mortality rate. CI, confidence interval; IV, inverse variance; VEGF, vascular endothelial growth factor.

**FIGURE 14:**
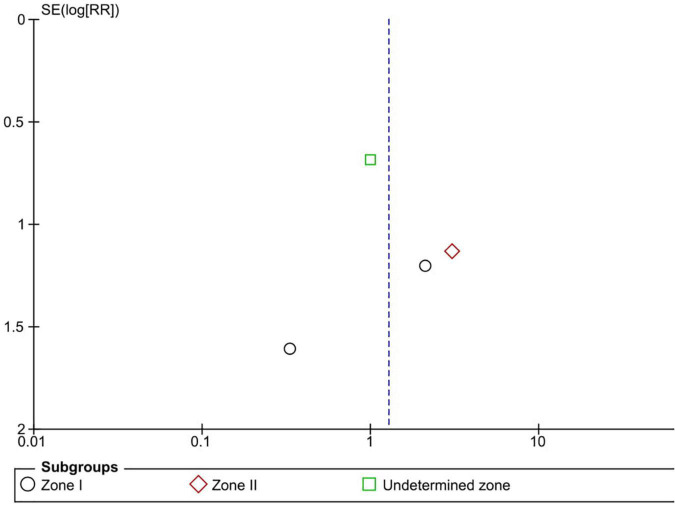
Funnel plot of mortality rate. SE, standard error; RR, risk ratio.

Six RCTs reported data on mortality (*n* = 565 infants) (1, 3, 10, 13, 15, 16). Intravitreal anti-VEGF injection showed similar mortality rates as retinal ablative therapy (RR = 1.28, 95% CI 0.48–3.41, *P* = 0.62, I^2^ = 0%). Similarly, there was no significant difference between the anti-VEGF injections and laser therapy in the zone I (RR = 1.10, 95% CI 0.17–7.20, *P* = 0.92, I^2^ = 0%), zone II (RR = 3.08, 95% CI 0.33–28.32, *P* = 0.32, I^2^ = not applicable), and undetermined zone (RR = 1.00, 95% CI 0.26–3.85, *P* = 1.00, I^2^ = not applicable) subgroups (Figure 13). The funnel plot was symmetric upon visual inspection; therefore, publication bias was unlikely (Figure 14). The GRADE certainty of evidence was found to be rated as moderate for mortality (Figure 6).

## Discussion

This systematic review and meta-analysis compared the efficacy and safety of intravitreal anti-VEGF monotherapy with laser therapy for treating ROP. The pooled effect estimate showed a statistically significant reduction in adverse events in favor of treatment with intravitreal anti-VEGF monotherapy. Nonetheless, no significant difference was found between the anti-VEGF injections and laser therapy with respect to recurrence, retreatment, treatment switching, and mortality.

In a retrospective study of 128 preterm infants with type 1 ROP, IVB, IVR, and laser were found to have low recurrence rates and be equally effective for ROP regression (17). Our results regarding zone I ROP match those of a recent systematic review in which anti-VEGF agents showed a lower recurrence rate than laser therapy in patients with zone I ROP (18). In another study, 82.9% of 70 eyes with zone I ROP regressed after a single IVB injection, showing that treatment with IVB monotherapy is effective for zone I ROP regression (1, 19). Similarly, ROP regressed after the first IVB injection in 95.4% of 238 eyes with pre-threshold, threshold, or aggressive posterior ROP (20). For more clarification, the significant reduction in recurrence rate in the anti-VEGF group -although this outcome rated as very low quality of evidence in our systematic review according to the GRADE criteria-, it goes in accordance with the guideline of the Royal College of Ophthalmologists (RCOphth), in which they stated a grade A evidence supporting the use of intravitreal anti-VEGF injections as a first-line treatment for eyes with zone I ROP (5). Despite the promising results, including low recurrence rates and ROP regression following IVB injections, a recent study reported a case of retinal neovascularization and ROP reactivation 10 years after successful treatment with IVB monotherapy for type 1 ROP. Therefore, long-term follow-up data should be considered when evaluating ROP recurrence (19, 21). A retrospective interventional case series of 12 infants (23 eyes) with a mean birth weight of 821.58 g (standard deviation = 297.63) found that a 0.25 mg IVR injection led to regression in all infants with stage 3 ROP, and none of the eyes needed additional treatment (22). Although decreasing the intravitreal VEGF level in ROP eyes is the therapeutic hallmark of treating ROP, discrepancies between the findings regarding recurrence or disease progression could be due to the different definitions used in staging ROP and discrepancies in defining ROP recurrence (23).

A meta-analysis of 3,701 eyes with ROP found that laser therapy was associated with a significantly lower likelihood of requiring supplementary treatment than anti-VEGF injections. However, data stratification by ROP zone was limited; hence, no solid conclusion could be drawn (4). A retrospective review of infants with type 1 ROP revealed that only 5.7% of infants required retreatment following IVB (24). This was also seen in another study, in which most patients who received retreatment initially had aggressive posterior ROP, also known as aggressive ROP (4, 25, 26). This could be explained by the fact that aggressive ROP has a severe nature distinguished by the rapid development of pathologic neovascularization and severe plus disease (26). In a recent cohort study, the likelihood of retreatment after laser therapy was 20.4% compared with 66.7% after anti-VEGF therapy, which confirms the need for careful and extensive long-term follow-up after intravitreal injections of VEGF inhibitors because delayed recurrence has been reported to occur up to 19 weeks or even 2 years after treatment (21, 27, 28). Our review showed no significant difference in treatment switching and retreatment rates between anti-VEGF injection and laser therapy in zone I and undetermined zone subgroups. Nevertheless, at zone II, treatment switching and retreatment were very prevalent in the anti-VEGF group. This could explain the recommendations of the RCOphth in their clinical guideline on the treatment of ROP, in which they recommended the use of transpupillary laser over intravitreal anti-VEGF injections for eyes with zone II ROP (5). Discrepancies between the studies could be due to different birth weights or differences in the indications for additional treatment.

Alterations in the anterior segment of the eye, resulting in very high myopia, were seen in the eyes of infants enrolled in the BEAT-ROP study who received retinal ablative therapy (51.4% zone I, 36.4% zone II) (29). Among 13 inborn infants with type 1 zone I ROP who received 0.5 mg IVB injection for one eye and laser therapy for the other eye, two eyes that received laser therapy progressed to retinal detachment, and at 9 months, all eyes receiving IVB had favorable anatomic outcomes yet showed some abnormalities on fluorescein angiography (15). In the RAINBOW study, mortality, adverse effects, and non-serious systemic adverse events were evenly distributed among those who received 0.2 mg IVR, 0.1 mg IVR, and laser therapy (10). The RAINBOW extension study reported the 2-year outcomes of the patients treated with 0.2 mg IVR and 0.1 mg IVR. The prevalence of high myopia was lower in the IVR 0.2 mg arm than in the laser arm. IVR 0.2 mg was found to be effective and safe in infants up to 2 years of age since no effects on growth, blood pressure, neurodevelopmental scores, or pulmonary manifestations were detected (30). Although our review showed that anti-VEGF agents are better and safer than laser in terms of adverse events, systemic side effects are difficult to assess. In contrast, another systematic review revealed that IVB treatment for severe ROP was associated with an increased risk of cognitive impairment and low cognitive and language scores in preterm infants (31). However, no systemic complications of IVR or aflibercept were reported (32, 33). The systemic complications of intravitreal anti-VEGF agents in adults are still unclear. Thus, uncertainty remains about the systemic toxicity of intravitreal VEGF inhibitors in infants (34, 35). At present, two RCTs (the FIREFLEYE and BUTTERFLEYE trials) comparing the efficacy and safety of intravitreal aflibercept and laser therapy are being conducted (36, 37). The binding affinity of aflibercept to the VEGF receptor is 100 times higher than that of ranibizumab and bevacizumab. Notably, aflibercept alone can inhibit VEGF and placental growth factors 1 and 2 (38–40). More pronounced suppression of systemic VEGF has been reported in ROP infants treated with IVB than in those treated with aflibercept. VEGF is essential for vascularization and homeostasis in the brain; thus, reduced VEGF levels would implicate systemic side effects in terms of intellectual function and neurodevelopment (41). Because of the abovementioned advantages, we eagerly await the results of the FIREFLEYE and BUTTERFLEYE trials as they could provide reliable data on aflibercept, which could change current practice. Specifically, aflibercept could become the treatment of choice if the forthcoming RCTs support the data of the published studies. Intravitreal aflibercept has been shown to be effective in inducing complete regression irrespective of ROP type (42). The lowest effective dose of anti-VEGF agents should be used when treating ROP to minimize complications. Although there is no consensus on the optimum dose of anti-VEGF in ROP treatment, the recommended bevacizumab dose for ROP infants is 0.625 mg.

More RCTs are warranted to determine the lowest sufficient dose of anti-VEGF for ROP treatment, the anti-VEGF agent to be used, and the optimal duration of follow-up (2, 9). Although the effectiveness and safety of intravitreal anti-VEGF agents in the management of ROP have been investigated, conflicting results and debate remain. To date, our systematic review and meta-analysis is the most comprehensive effort to consolidate published findings of RCTs. Additionally, it had a relatively large sample size and included RCTs with high levels of evidence. Most of the included RCTs were well conducted and had an overall low risk of bias. Furthermore, subgroup analysis of the ROP zones was performed, which improves the clinical relevance. Finally, this high-quality systematic review and meta-analysis provides the GRADE criteria for each of the studied outcomes. The GRADE criteria take into consideration five major domains, namely, risk of bias, imprecision, inconsistency, indirectness, and publication bias, and three other domains, namely, magnitude of effect, dose response, and confounding. Outcomes evaluation using such criteria ensures transparent assessment of the certainty of evidence with an explicit and comprehensive evaluation of the outcomes pertaining to alternative management strategies. This enables us to provide reliable and pragmatic recommendations. Although the GRADE approach enables confident determination of the quality of evidence, it does not eliminate the need for clinical judgment. We believe that no systematic review on the safety and efficacy of anti-VEGF monotherapy in infants with ROP has used the GRADE criteria.

The review has some limitations. First, obvious variability was present in anti-VEGF doses, gestational age, and birth weight across the included RCTs, which might affect the results drawn from the studies. Second, the studies included in this meta-analysis showed lots of heterogeneity, probably secondary to variability in the patient populations and treatment protocols. Third, risk of bias was found in some studies, especially related to the randomization technique, deviation from the intended intervention, and selection of the reported results. All these limitations resulted in moderate to very low quality of evidence in most of the investigated outcomes, except for the adverse event outcome which was rated as high quality.

## Conclusion

Overall, intravitreal anti-VEGF monotherapy was associated with fewer adverse events than laser therapy, rated as high quality of evidence according to the GRADE criteria. Pooled analysis revealed no significant difference between the two arms with respect to the recurrence rate, treatment switching, retreatment, and mortality, with quality of evidence ranging from moderate to very low as per the GRADE approach. As per the ROP zone stratification, anti-VGEF monotherapy was associated with a significantly lower recurrence rate and fewer adverse events compared to laser therapy for eyes with zone I ROP. At zone II, anti-VEGF monotherapy was associated with significantly higher retreatment and treatment switching rates, yet fewer adverse events compared to retinal ablative therapy. Nevertheless, practice-changing clinical recommendations cannot be concluded due to the low quality of most of the studied outcomes evidence per the GRADE criteria. Further high-quality RCTs are warranted before making formal clinical recommendations about the superiority of anti-VEGF agents or laser therapy in the clinical practice in treating ROP. Additionally, more well-designed studies are required to examine the long-term systemic side effects of anti-VEGF agents, investigate the effects of different intravitreal anti-VEGF agents on different ROP zones and stages, and examine the efficacy and safety of different doses of anti-VEGF agents. Moreover, consensus on the definitions of ROP recurrence and ROP requiring retreatment is needed as variability hinders the generalization of results corresponding to each ROP zone.

## Data Availability Statement

The original contributions presented in the study are included in the article/Supplementary Material, further inquiries can be directed to the corresponding author/s.

## Author Contributions

NT, AG, and SA-G contributed to the research idea, design of the study, data extraction, statistical analysis/interpretation, risk of bias assessment, and writing/editing of the final manuscript. JH and BA-H contributed to the design of the study, data extraction, statistical analysis and interpretation, and writing/editing of the final manuscript. LA contributed to the data extraction, statistical analysis/interpretation, risk of bias assessment, and writing/editing of the final manuscript. HA contributed to the statistical analysis/interpretation and writing/editing of the final manuscript. All authors contributed to the article and approved the submitted version.

## Conflict of Interest

The authors declare that the research was conducted in the absence of any commercial or financial relationships that could be construed as a potential conflict of interest.

## Publisher’s Note

All claims expressed in this article are solely those of the authors and do not necessarily represent those of their affiliated organizations, or those of the publisher, the editors and the reviewers. Any product that may be evaluated in this article, or claim that may be made by its manufacturer, is not guaranteed or endorsed by the publisher.
