# Supplementary material for: Intravitreal Anti-vascular Endothelial Growth Factor Injection for Retinopathy of Prematurity: A Systematic Review and Meta-Analysis
Source: Front Med (Lausanne). 2022 May 9;9:884608. doi: 10.3389/fmed.2022.884608 (PMC9124790; doi:10.3389/fmed.2022.884608)
Supplement: Supplementary file 1 [file Data_Sheet_1.docx]

Supplementary Appendix

Intravitreal Anti‐Vascular Endothelial Growth Factor Injection for Retinopathy of Prematurity: A Systematic Review and Meta-analysis

Nada O Taher, Abdullah A Ghaddaf, Sarah A Al-Ghamdi, MD, Jumanah J Homsi, MD, Bandar J Al-Harbi, MD, Lugean K Alomari, Hashem S Al-Marzouki, MD

Search strategy for Medline, Embase, and Cochrane Central Register of Controlled Trials (CENTRAL), last performed on **15/7/2021** (n=422)

1. exp "Retinopathy of Prematurity"/

2. Retrolental fibroplasia$.mp.

3. Retinopathy of prematurity$.mp.

4. 1 or 2 or 3

5. exp Bevacizumab/

6. Bevacizumab$.mp.

7. 5 or 6

8. exp Ranibizumab/

9. Ranibizumab$.mp.

10. 8 or 9

11. Aflibercept$.mp.

12. Pegaptanib$.mp.

13. 7 or 10 or 11 or 12

14. exp Clinical Trial/ or exp Controlled Clinical Trial/ or exp Randomized Controlled Trial/

15. Trial$.mp.

16. 14 or 15

17. 4 and 13 and 16

18. exp retrolental fibroplasia/

19. 4 or 18

20. 13 and 16 and 19
